# Folic acid intervention during pregnancy alters DNA methylation, affecting neural target genes through two distinct mechanisms

**DOI:** 10.1186/s13148-022-01282-y

**Published:** 2022-05-16

**Authors:** Miroslava Ondičová, Rachelle E. Irwin, Sara-Jayne Thursby, Luke Hilman, Aoife Caffrey, Tony Cassidy, Marian McLaughlin, Diane J. Lees-Murdock, Mary Ward, Michelle Murphy, Yvonne Lamers, Kristina Pentieva, Helene McNulty, Colum P. Walsh

**Affiliations:** 1 Genomic Medicine Research Group, Ulster University, Coleraine, Northern Ireland UK; 2 Nutrition Innovation Centre for Food and Health (NICHE), School of Biomedical Sciences, Ulster University, Coleraine, Northern Ireland UK; 3 Psychology Institute, Ulster University, Coleraine, Northern Ireland UK; 4 Unitat de Medicina Preventiva i Salut Pública, Facultat de Medicina i Ciències de La Salut, Universitat Rovira i Virgili, Reus, Spain; 5 Food, Nutrition, and Health Program, Faculty of Land and Food Systems, The University of British Columbia, and British Columbia Children’s Hospital Research Institute, Vancouver, BC Canada; 6 Centre for Research and Development, Region Gävleborg/Uppsala University, Gävle, Sweden; 7 Present Address: The Johns Hopkins University School of Medicine, Baltimore, USA

**Keywords:** Folic acid, DNA methylation, Neurodevelopment

## Abstract

**Background:**

We previously showed that continued folic acid (FA) supplementation beyond the first trimester of pregnancy appears to have beneficial effects on neurocognitive performance in children followed for up to 11 years, but the biological mechanism for this effect has remained unclear. Using samples from our randomized controlled trial of folic acid supplementation in second and third trimester (FASSTT), where significant improvements in cognitive and psychosocial performance were demonstrated in children from mothers supplemented in pregnancy with 400 µg/day FA compared with placebo, we examined methylation patterns from cord blood (CB) using the EPIC array which covers approximately 850,000 cytosine–guanine (CG) sites across the genome. Genes showing significant differences were verified using pyrosequencing and mechanistic approaches used in vitro to determine effects on transcription.

**Results:**

FA supplementation resulted in significant differences in methylation, particularly at brain-related genes. Further analysis showed these genes split into two groups. In one group, which included the *CES1* gene, methylation changes at the promoters were important for regulating transcription. We also identified a second group which had a characteristic bimodal profile, with low promoter and high gene body (GB) methylation. In the latter, loss of methylation in the GB is linked to decreases in transcription: this group included the *PRKAR1B*/*HEATR2* genes and the dopamine receptor regulator *PDE4C*. Overall, methylation in CB also showed good correlation with methylation profiles seen in a published data set of late gestation foetal brain samples.

**Conclusion:**

We show here clear alterations in DNA methylation at specific classes of neurodevelopmental genes in the same cohort of children, born to FA-supplemented mothers, who previously showed improved cognitive and psychosocial performance. Our results show measurable differences at neural genes which are important for transcriptional regulation and add to the supporting evidence for continued FA supplementation throughout later gestation. This trial was registered on 15 May 2013 at www.isrctn.com as ISRCTN19917787.

**Supplementary Information:**

The online version contains supplementary material available at 10.1186/s13148-022-01282-y.

## Background

Folate is essential for cell division and tissue growth, and the requirement for this B vitamin particularly increases during pregnancy due to its important role in embryonic development. Conclusive evidence from randomised controlled trials (RCTs) has proved the clear benefits of peri-conceptional supplementation with FA, the synthetic form of folate, against neural tube defects (NTD)[1, 2]. As a result, women worldwide are recommended to take 400 μg FA/day from preconception until the end of the first trimester to prevent NTD-affected pregnancies. While the first trimester is a significant time for neural tube closure and the formation of brain vesicles, in second and third trimester there occurs massive expansion of the brain accompanied by synaptogenesis and cortex maturation: thus continued maternal supplementation could have benefits beyond the prevention of NTDs [3–6]. These developmental and histological changes are accompanied by significant changes to the epigenome, in particular DNA methylation, and FA is a major component of the methyl donor cycle (reviewed in [5]). DNA methylation is known to control gene expression across different gene classes, including imprinted genes (many of them expressed in the brain), genes on the inactive X, germline genes and retrotransposons [7].

While DNA methylation within the promoter or enhancer of genes is associated with transcriptional repression, when located within the gene body (GB) it can promote expression [8, 9]. DNA methylation is important for embryonal development as mouse embryos with DNMT mutations do not survive birth [10, 11]. Many observational studies also report DNA methylation alterations of genes involved in neurodevelopmental disorders [12, 13]. Changes in DNA methylation at multiple loci have been linked with NTDs and fragile X-syndrome [14–17], where folate plays a direct preventive role. Methylation defects are also seen in imprinting disorders with a neurological component such as Prader–Willi syndrome and Angelman syndrome, as well as in disorders such as Rett syndrome [18, 19] and autism spectrum disorder [20, 21]. There has been growing evidence of such brain-related genes being controlled by GB methylation in both mice and humans [8, 22, 23].

**Fig. 1:**
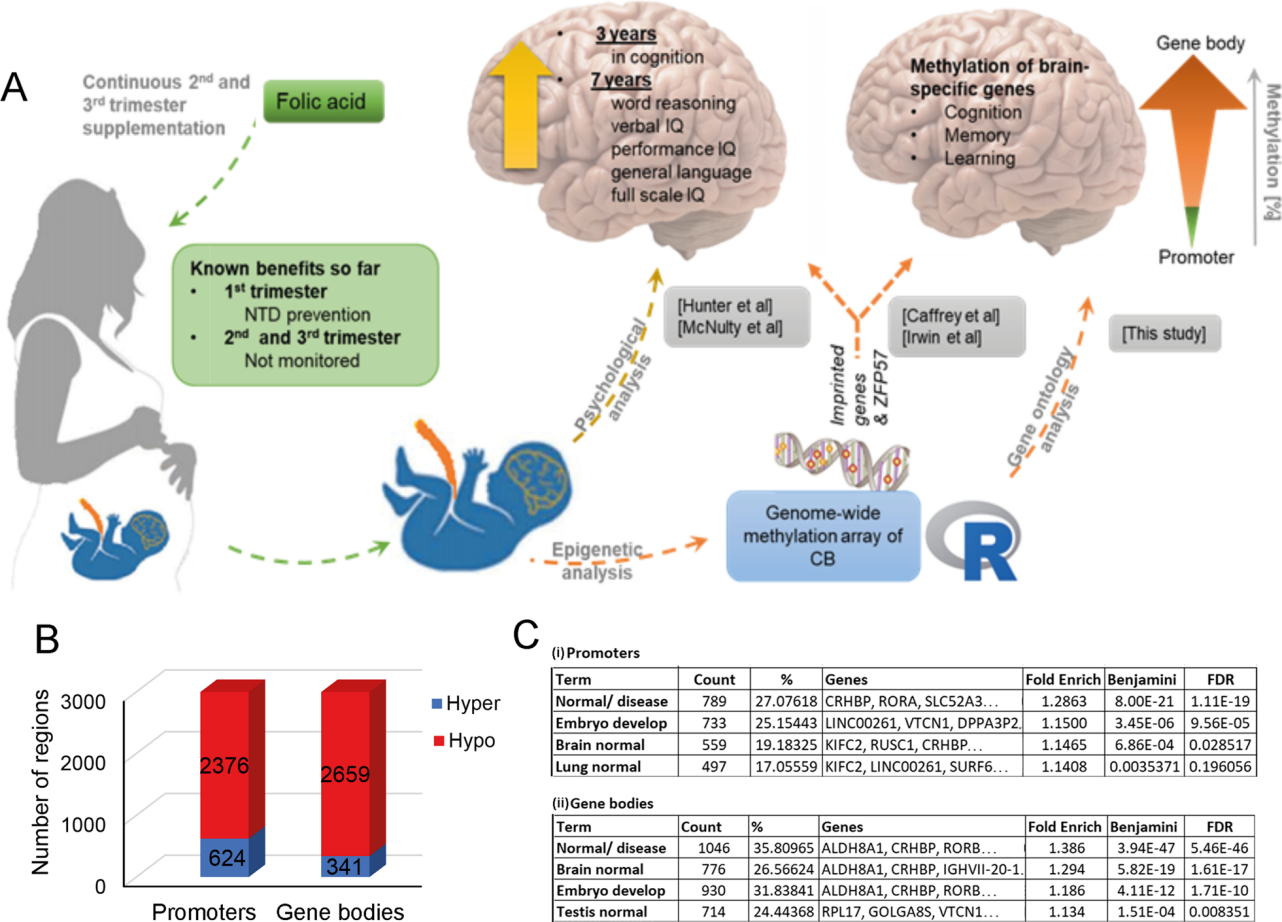
Folic acid methylation changes are particularly enriched at brain-specific genes. **A** Overview of FA role in DNA methylation-driven benefits for child. Schematic of the EpiFASSTT study and already published results and summary of the results found in this study (grey). Folic acid affects nutritional outcomes for mother and child in the form of maintaining folate levels (green). Psychological analysis of the children supplemented with FA shows clear cognitive benefits (brown arrow). Methylation analysis of the CB samples associates methylation changes with brain-related gene groups (orange). **B** Number of hyper- (blue) and hypomethylated (red) regions (sites, promoters, and genes) in the top 3000 best-ranking promoters and gene bodies in response to response to FA supplementation in later pregnancy. **C** Enrichment for tissue-type expression using DAVID and Unigene for the top 3000 best-ranking promoters (i) and gene bodies (ii). Term, Unigene terms; Count; number of genes from the input set in this category; %, per cent of total input; Genes, names of genes from input set falling into this category (first 3 only shown); Fold Enrich, fold enrichment; Benjamini, *p* value corrected for multiple testing by Benjamini–Hochberg method; *FDR* false discovery rate score, where 0.05 = 5% of hits being false positive. A significant percentage of promoters and genes changing methylation are expressed in the brain and during early development

Both animal [3, 24] and observational human studies showed that FA levels affect brain function, particularly cognition [25], motor skills [26], language and communication [27, 28]. Notably, we also recently reported results from the FASSTT trial where mothers with singleton pregnancies, who took FA during the first trimester, were randomised at the 14th gestational week (GW) and split into placebo and FA (400 μg/day) groups for trimesters two and three (see Fig. 1A). Standardised psychological tests of the children at age 3 and 7 years showed higher verbal IQ, performance IQ, general language, and full-scale IQ for the supplemented group when compared with a nationally representative sample of British children the same age [29]. In addition, these children showed significantly elevated scores on the Resiliency Attitudes and Skills Profile (RASP) and Trait Emotional Intelligence Questionnaire Child Short Form (TEIQue‐CSF) [30]. Most recently, we showed more efficient semantic processing of language (demonstrated using magnetoencephalographic brain imaging) in the same cohort at 11 years [31]. Our studies to date have not fully addressed, however, the biological mechanisms underpinning the relationship of maternal FA exposure during pregnancy with neurodevelopment.

We previously carried out DNA methylation analyses on this cohort and found both alterations at multiple imprinted loci [32] and at a region close to the zinc finger protein 57 (*ZFP57*) locus, which regulates imprinting [33]. While the latter helped to explain the effects on imprinting, the association with higher-order cognitive function was limited. Here, we have used the EPIC array results to examine functionally related groups of genes where FA-mediated methylation changes occur. We report here that a high proportion of affected genes are associated with brain function through gene ontology (GO) analysis, with multiple individual promoters and genes being affected. We verified our findings using pyrosequencing in CB and our newly released online *CandiMeth* workflow in Galaxy [34], which allows mapping, visualisation, and quantification of methylation changes in a user-friendly, non-command line environment. These findings report novel associations between FA supplementation in later pregnancy, improved cognitive performance and epigenetic alterations at genes involved in higher brain function.

## Results

### Folic acid supplementation caused hypomethylation particularly at brain-related genes

Analysis of the genome-wide methylation results from the Infinium MethylationEPIC Beadchip Array [33] was carried out on the CB samples from the FA-treated and untreated cohorts. Raw data was analysed using the *RnBeads* package and results are presented as mean *β* values varying from 0 to 1, with 0 being unmethylated and 1 fully methylated. Given the number of study participants and the nature of the intervention, we looked at differentially methylated regions (DMR) rather than individual sites, as false discovery rate (FDR)-corrected *p* values were not significant, and as we [33, 35] and others [36] have shown consistently better performance in identifying biologically significant changes using this approach. *RnBeads* looks for contiguous sites showing concerted changes, and ranks these regions based on a combination of the change in mean methylation, the quotient of mean methylation, and the combined *p* value to account for not only the statistical significance, but also the magnitude of methylation change. The analysis of the list of top 3000 best ranking promoters found most (*n* = 2376/3000) lost methylation (Fig. 1B): the same was true of GB (*n* = 2659/3000). In order to determine if there was enrichment for any type of gene, we input these lists into the bioinformatics analysis suite DAVID [37, 38]. DAVID allows the user to objectively score trends in gene enrichment under a variety of headings, including cellular location of the gene product, biological process, and tissue expression pattern. The initial analysis indicated that the most statistically significant type of enrichment overall was for tissue-type expression, scored using the Unigene database, as these had the lowest *p* values following correction for multiple testing using Benjamini–Hochberg correction and the best false discovery rate (FDR). This further showed that of the hypomethylated promoters (Fig. 1C (i)), those for genes expressed in brain were the third most-enriched category (count 559, FDR = 0.029) and brain was the second most-enriched term for GB (count 776, FDR = 1.61 × 10^−17^—Fig. 1C(ii)). These results suggested that the substantial methylation differences seen between treated and untreated samples, when measured in cord blood (CB), included a strong effect on genes involved in neural function.

We next looked at those regions showing most robust differences in methylation between the two groups. Table 1 presents the 10 top-ranking promoter regions from *RnBeads*: discounting the pseudogenes (*ANKRD20A11P*, *PTCHD3P2*, and *CNOT6LP1*), whose function—if any—is unclear, 6/7 of the remaining promoter regions were either expressed in the brain or associated with neurodevelopmental phenotypes, as indicated in the table which includes references to the relevant publications. The region with the best combined rank, i.e. the smallest value, was the imprinted gene regulator *ZFP57* (already examined in detail in [33]). Among other promoters affected by FA was that of the *CES1* gene, previously associated with Down’s syndrome (DS) (loss of 3.86% methylation in the treatment group vs. placebo); *DUSP22* which shows alterations in schizophrenia (gained 6.24%); and the microRNA locus *MIR4520A*/*B* (lost 7.22%) which also showed changes in methylation in young adults with depression. Two further promoters with smaller changes were *HEATR2*, which is mutated in ciliary dyskinesia (lost 2.23%), and the dopamine signalling-related gene *PDE4C* (lost 2.04%), although the latter only comprised one probe representing one CG (confirmed not to be a known single-nucleotide polymorphisms (SNP)). The only remaining target with no known tie to brain or behaviour was the antiretroviral gene *TRIM6-TRIM34* with a loss of methylation of 3.77%. Overall, the analysis showed strong ties between FA supplementation and genes related to brain function and neurodevelopmental phenotypes.

**Table 1:** Top 10 promoter regions with differential methylation show links to neurodevelopmental phenotypes

Promoter^a^	Full name	# CG sites	Chr.^b^	Position	Mean diff (%)^c^	Rank^d^	Comb. p val^e^	Function/clinical correlates	Reference
**ZFP57**	Zinc finger protein 57	15	chr6	29648388–29650387	6.23	79	0.011946	Maintains methylation at imprinted genes, many expressed in brain	[82, 83]
[ANKRD20A11P]	Ankyrin repeat domain 20 family, A11, pseudogene	10	chr21	15352259–15354258	3.32	118	0.007401	Pseudogene	–
**CES1**	Carboxylesterase 1	8	chr16	55866750–55868749	− 3.86	154	0.010683	Hydrolysis or transesterification of various xenobiotics. Hypermethylated in Down syndrome	[43]
**DUSP22**	Dual specificity phosphatase 22	6	chr6	290130–292129	6.24	192	0.027263	Activator of the JNK signalling pathway. Hypermethylation seen in famine-exposed schizophrenia patients	[73]
**MIR4520A; MIR4520B**	MicroRNA 4520a/4520b	7	chr17	6558329–6560328	− 7.22	205	0.02862	Hypermethylated in young adults with depression	[35, 84]
**HEATR2/PRKAR1B**	HEAT repeat containing 2/protein kinase, cAMP-dependent, regulatory, type I, beta	11	chr7	764838–766837	− 2.23	208	0.015631	HEATR-assembly of cilia found in lung & brain; PRKAR1B-expressed in brain, associated with neurodegenerative disorders	[39, 62, 85]
[PTCHD3P2]	Patched domain containing 3 pseudogene 1	1	chr2	170624810–170626809	− 3.69	210	0.029302	Pseudogene	–
**PDE4C**	Phosphodiesterase 4C, cAMP-specific	1	chr19	18365730–18367729	− 2.05	270	0.009722	Regulation of dopamine D1A receptor signalling; gained methylation in response to B-vit in an RCT w/elderly	[46, 86]
[CNOT6LP1]	CCR4-NOT transcription complex, subunit 6-like	51	chr15	56298875–56300874	− 3.98	293	0.036665	Pseudogene	–
TRIM6-TRIM34	TRIM6-TRIM34 readthrough	10	chr11	5616455–5618454	− 3.77	336	0.039767	Antiretroviral genes	[87, 88]

^a^Genes with neural links in bold, pseudogenes in square brackets; ^b^Chr, chromosome; ^c^mean.diff (%), difference in mean *β* value expressed as %, ^d^Rank, RnBeads computed ranking value (lowest being best); ^e^comb. *p* val, combined *p* value

Whilst analysis by genomic regions is generally accepted as being more robust, for completion we also examined the top 10 best ranking sites (Additional file 2: Table S1). Two of the CG sites were located in the intron of a thioredoxin superfamily gene *NXN*, associated with Robinow syndrome (a rare disorder with limb shortening and abnormalities of the head and face), and were previously confirmed by us using pyrosequencing [33]. Two of the other sites were located within gene *PRKAR1B*, mutated in a neurodegenerative disorder [39] and which lies head-to-head with the *HEATR2* gene reported in Table 1. Apart from *NXN* and *PRKAR1B*, other genes had only one CG site and so are less likely to represent significant changes. One of these *SEMA4F* was related to neural development [40] and showed a 9% loss of methylation. Other sites included probes outside the immune response gene *RAD51B*, the testis-expressed *AK125858* [41], and the *CYP4V* gene. The CG sites in the *ATP11A* and *MAGI2* genes each contained rarer SNPs left out by the initial quality control processing, which led to the appearance of a change in methylation and can be discounted.

Considering that the highest proportion of regions showing hypomethylation were GB, we also looked at the top-ranking regions in that category (Additional file 2: Table S2). However, due to the larger interval sizes and inclusion of introns, many of the hits were less informative, with 5 of the top 10 being pseudogenes (*PTCHD3P2*, *USP32P2*, *ALOX15P2*, *ZNF726*, and *IMMTP1*), notably though the *MIR4520A*/*B* and *ZFP57* loci (already presented in Table 1) also appeared in the top 10 regions here. Other genes with small magnitude changes were two micro-RNAs without known function (*MIR648* and *MIR4740*) and the DNA binding factor *FOXD4L5.*

### Activation of *CES1* by promoter demethylation in in vitro models

**Fig. 2:**
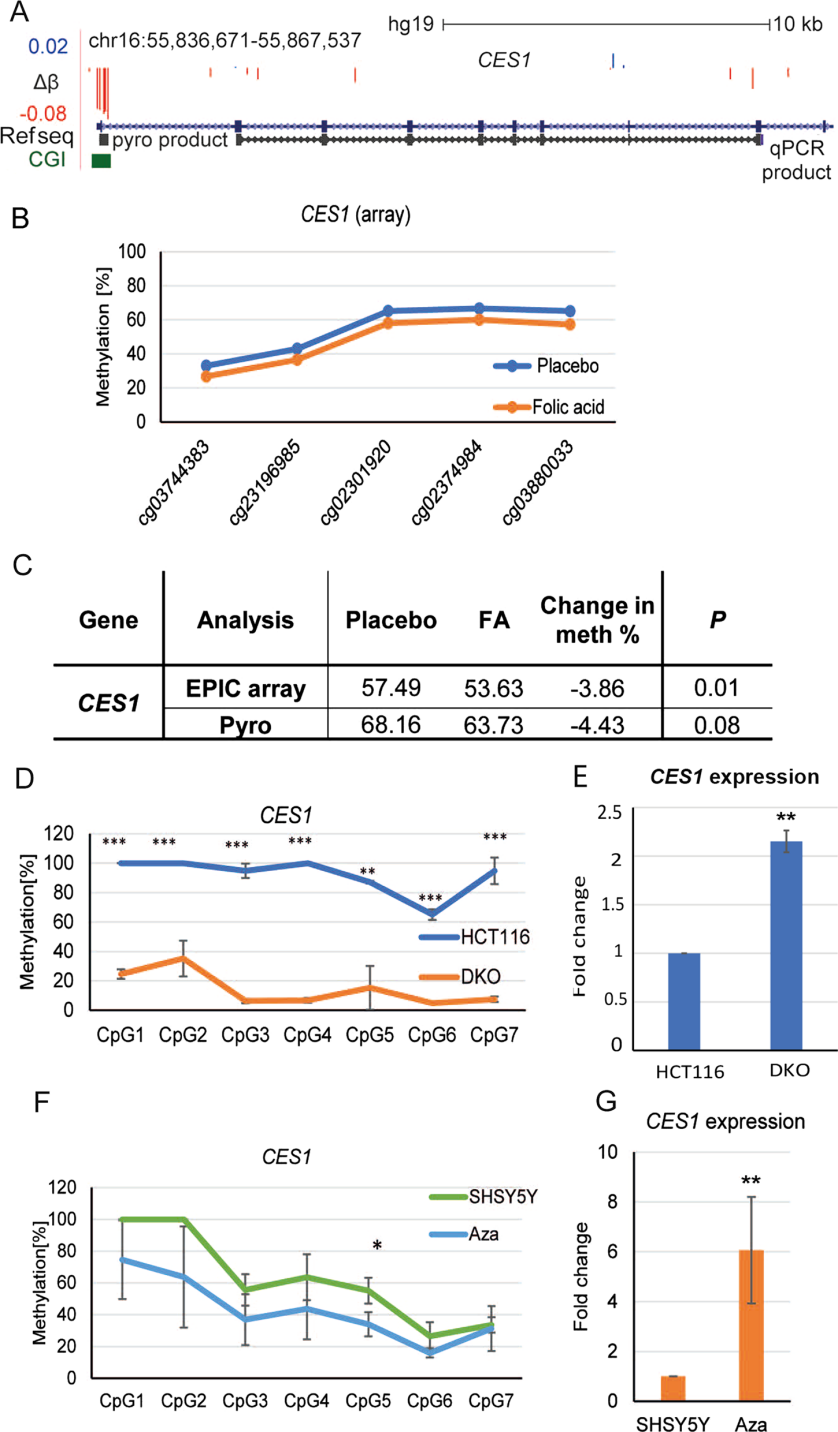
Promoter methylation of *CES1* in response to FA supplementation. **A** Schematic of *CES1* gene in UCSC browser with genomic coordinates in hg19 genome release. EPIC array probes are shown at the top; maximum gain and loss for *β* is also shown (+ 0.02*β* = 2%, − 0.08*β* = 8% methylation). Corresponding introns (blue lines with arrows marking the direction of transcription) and exons (blue rectangles) of the gene are marked. The position of the CGI within the region is highlighted (green). Pyro assay product used for DNA methylation analysis in the CB samples and cell lines (black) and RT-qPCR product (black rectangles presenting exons and lines with arrows-spliced introns marking orientation of the PCR reaction) used for assessing gene expression in cell lines are shown in track at the bottom. **B** *CES1* promoter loses methylation after FA supplementation. Mean methylation percentage within highlighted CG sites in FA-treated and placebo CB samples by EPIC array. **C** Confirmation of mean DNA methylation of *CES1* promoter within region shown in **A** comparing EPIC array and pyrosequencing. Placebo, FA-mean average of the individual means in the placebo and FA-treated groups; change of methylation–methylation difference between the groups; *P*-probability by Student’s *t* test. **D** Methylation levels at individual CG sites covered by the pyroassay in WT (HCT116, blue) and knockout (DKO, orange) cells. Values are shown as mean +/− SD for each site: *p < 0.05; **p < 0.01; ***p < 0.001. **E** RT-qPCR showing upregulation of gene in HCT116/DKO cells using the primers indicated in **A**, values normalized to *HPRT*. **F** Methylation levels using pyroassay as in D but in SHSY5Y (WT, green) compared to aza-treated SH-SY5Y cells (Aza, blue). Values are shown as mean +/− SD for each site: *p < 0.05; **p < 0.01; ***p < 0.001. **G** Upregulation of gene expression by RT-qPCR in aza-treated SH-SY5Y cells. All values are shown as mean +/− SD for each site or cell line: *p < 0.05; **p < 0.01; ***p < 0.001

We next looked in more detail at those promoter regions highlighted (Table 1) by the genome-wide scan. The carboxylesterase *CES1 is* involved in cocaine metabolism [42], and hypermethylation of the promoter has been reported in patients with Down’s syndrome [43]. The identified promoter included five CG sites across a region of approximately 300 bp at the first exon, overlapping a CGI. Figure 2A shows a genomic map of the locus overlaid with a track showing the locations of EPIC array probes and whether they gained or lost methylation in response to FA supplementation. Also shown is a graph of averaged methylation values at each CG probe across the promoter region, showing a clear loss of methylation across all sites in response to FA (Fig. 2B). To verify the hypomethylation using another method, we chose a methylation-sensitive pyrosequencing assay (pyroassay) as before. Due to technical limitations resulting from the CG density of the region, we were only able to cover two CGs corresponding to array probes (cg02374984 and cg02301920), but the overall loss of methylation agreed well with the array results and showed 4.43% loss (*p* = 0.08) compared to 3.86% loss for array (*p* = 0.01) (Fig. 2C).

We then wished to test mechanistically if such differences could impact on transcription from the cognate gene. To do this, we initially used the well-characterised pair of colorectal cancer cell lines HCT116 wild type (WT) and double knockout (DKO), which have high methylation levels at most loci in WT and low levels in the daughter cell line DKO due to decreased levels of *DNMT1* and *DNMT3B* enzymes [44]. Pyrosequencing showed > 90% methylation in WT HCT116 which dropped to ~ 20% in DKO (Fig. 2D). We then designed primers to cover part of the transcript as shown in Fig. 2A (qPCR product): reverse transcription of the mRNA and quantitative RT-PCR showed significantly increased transcription in the demethylated DKO cells compared to WT HCT116 (*p* < 0.01) (Fig. 2E).

To assess the potential role of epigenetic control of *CES1* promoter in neural cells as well, we used the neuroblastoma cell line SHSY5Y, which we treated with the DNA methyltransferase inhibitor 5-aza-2′-deoxycytidine (Aza). Methylation levels were high at most 5’ sites (100%) and decreased towards the 3’ end of the region (20%) in untreated cells (Fig. 2F); however, treatment with Aza resulted in loss of methylation at all but the most demethylated sites across the region. While differences in methylation only reached significance at one CG site (*p* = 0.03) due to variability in cell growth and drug uptake, treatment was clearly effective as qPCR confirmed a substantial and reproducible increase in transcription from *CES1* (Fig. 2G, *p* = 0.01). These data together showed that CGI-promoter-driven methylation was indeed important for transcriptional control of this neural gene.

### Confirmation of methylation changes at the *DUSP22* and *MIR4520A*/*B* loci

Two further regions whose methylation is also altered in schizophrenia (*DUSP22*) and depression (*MIR4520A*/*B*) were among the top-scoring promoters from the array analysis (Table 1). Like *CES1*, the *DUSP22* promoter contains a CGI (Additional file 1: Fig. S1A) and seven of the CGs in this region showed increased methylation (Table 1) as a response to FA supplementation. A pyroassay (Additional file 1: Fig. S1A) covering four of these CG sites showed a 6.93% increase in methylation in FA-treated CB samples (*p* = 0.01, treated *vs.* placebo), similar in magnitude and direction to the same four sites on the array (average gain 8.25%, *p* = 0.03 treated vs. placebo, see Additional file 1: Fig. S1C). To test for effects on transcription, we again used HCT116 and DKO cells: while the latter showed significantly lower methylation levels across the DUSP22 promoter (Additional file 1: Fig. S1D), there was no accompanying change in transcription for the transcript (Additional file 1: Fig. S1E).

For *MIR4520A*/*B*, the promoter region identified by *RnBeads* (Table 1) covered 2000 bp containing seven CG sites and overlapped two miRNAs *MIR4520A* and *MIR4520B* and showed 7.22% hypomethylation in the treatment group. Moreover, the same region was highlighted as a best ranking GB with average hypomethylation in the treated group being 12.2% (Additional file 2: Table S2). Due to pyrosequencing limitations and the CG probes covering the promoter being far apart, we were able to cover only one CG site located in the *MIR4520A*/*B* (pyrosequencing product shown in Additional file 1: Fig. S1F) but were able to confirm significant methylation loss within that site cg08750459 (*p* = 0.003/0.006) (Additional file 1: Fig. S1G, H).

### Hypomethylation at the CGI promoter for the divergently transcribed brain genes *PRKAR1B* and *HEATR2*

**Fig. 3:**
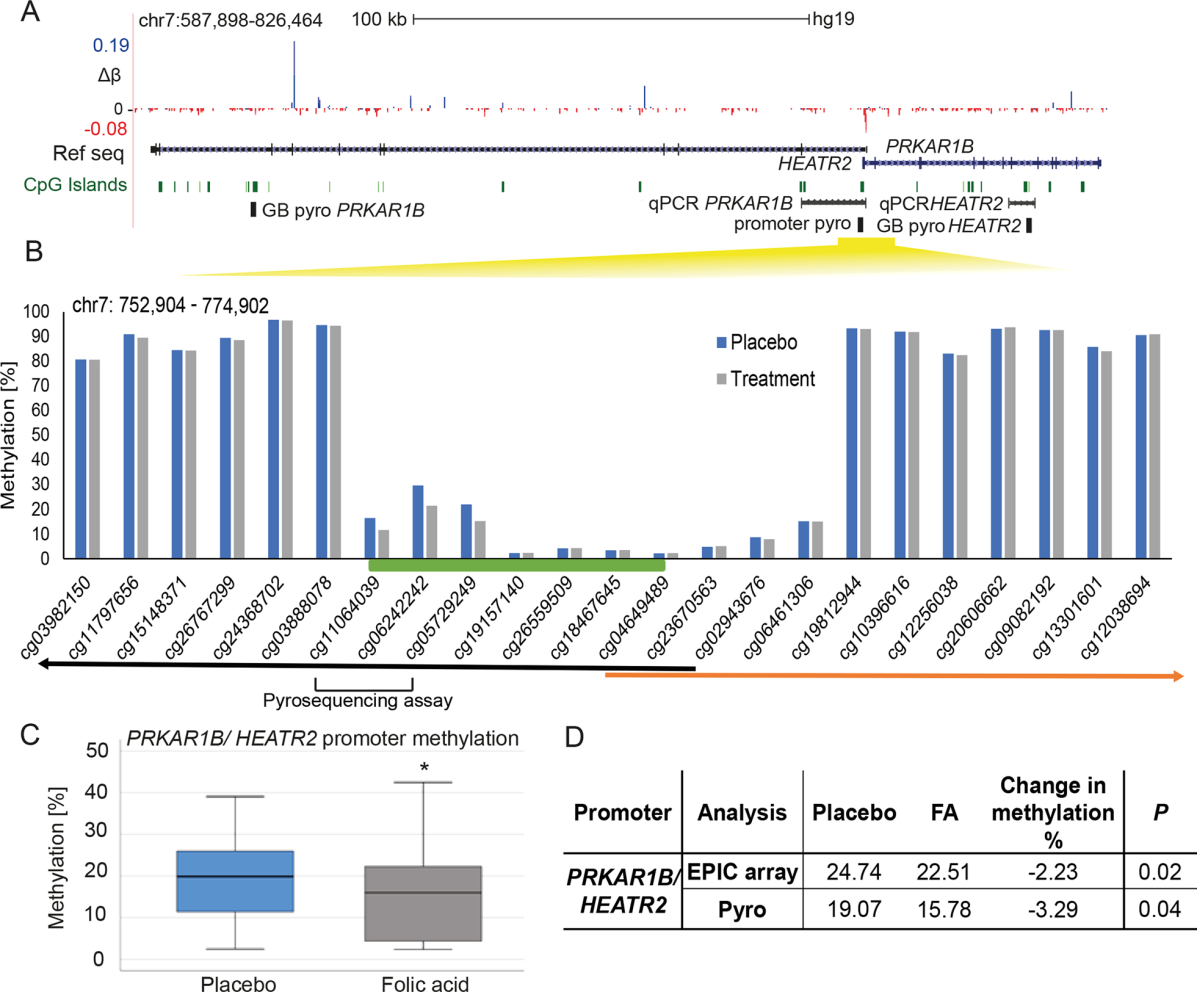
Methylation at the shared promoter for the *PRKAR1B* and *HEATR2* genes. **A** UCSC Genome browser tracks showing the region around the differentially methylated promoter of the divergently transcribed genes *PRKAR1B* and *HEATR2*, with genomic coordinates in hg19 human genome release, and scale as shown. EPIC array probes showing differential methylation (blue, gain; red, loss) are indicated, with size indicating the magnitude of change. The position of the pyrosequencing assay of promoter and GB (Pyro promoter/pyro GB) and RT-qPCR are also shown (black). Δ*β*, mean difference in *β* value between placebo and FA-treated groups; maximum gain and loss also shown (+ 0.19*β* = 19%, − 0.08*β* = 8% methylation). The position of the CGI within the region is highlighted (green lines). Both genes are transcribed from one promoter located within CGI (yellow bar). **B** Low promoter methylation overlapping CGI is accompanied by high GB methylation of *PRKAR1B* and *HEATR2*. Genome-wide methylation analysis of CB samples confirmed low levels of methylation at the promoter CGI (green bar) and high levels throughout the genes of both placebo (blue) and FA supplemented participants (grey). The location of indicative CGs assayed is shown as well as the direction of transcription from promoter CGI (green bar) of *PRKAR1B* (black arrow) and *HEATR2* (orange arrow). **C** FA supplementation affects the methylation of the shared promoter. Boxplot showing difference in methylation from pyroassay covering promoter shown in Table 1/Fig. 3A. **D** Average methylation at the promoter of *PRKAR1B*/*HEATR2* genes by pyrosequencing in the placebo and FA CB samples compared to EPIC 850 k array; *P* probability by Student’s *t* test

Amongst the top ten differentially methylated promoters was that for *PRKAR1B* (Table 1), which also contained two of the top ten differentially methylated sites (Additional file 2: Table S1). This promoter is located at a CGI, which also acts as the start for the divergently transcribed *HEATR2* gene (Fig. 3A, B—CGI in green). DNase I sensitivity (ENCODE) tracks available on the UCSC browser confirmed an open chromatin region within this promoter in several cell types (not shown). DNA methylation of an approximately 2 kb region was low around this region normally, consistent with the behaviour of most CGI, with high levels of methylation on both flanks (Fig. 3B).

As methylation at the CGI sites was relatively low, we wished to confirm the loss seen in response to FA using a second method, for which we again used pyroassays, targeted here at the promoter region (Fig. 3A, C). We were able to confirm the loss of methylation within the promoter in response to FA supplementation, in good agreement with the EPIC 850 k array in both direction and magnitude (array − 2.23%, *p* = 0.02; pyrosequencing − 3.29%, *p* = 0.04) (Fig. 3D).

### Demethylation facilitates transcription of *PRKAR1B* and *HEATR2*

**Fig. 4:**
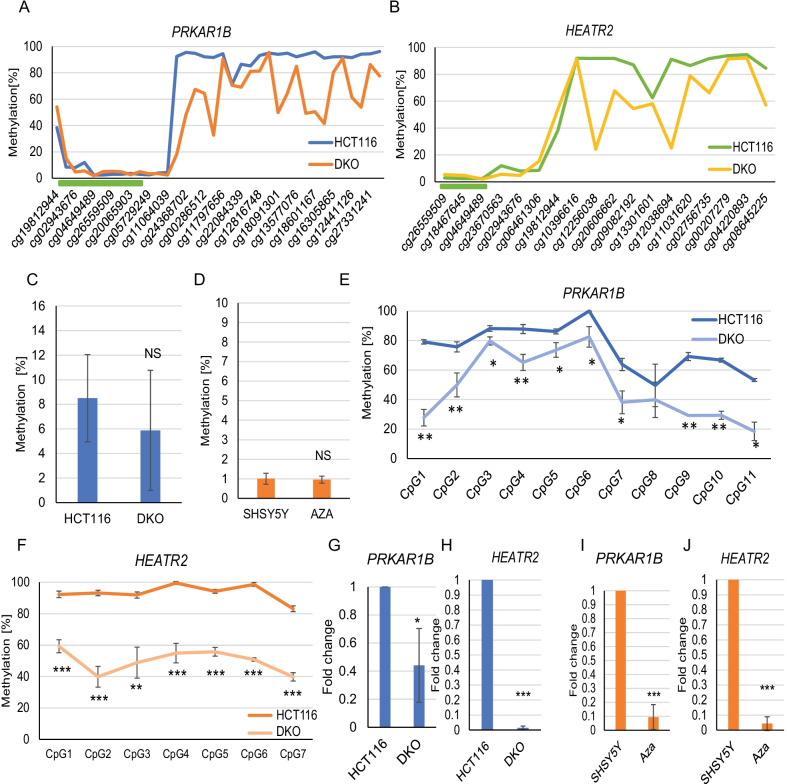
Gene body methylation drives expression of *PRKAR1B*/*HEATR2*. **A** Methylation levels at individual CG sites covered by the Illumina 450 K array in WT (HCT116, blue) and knockout (DKO, orange) cells within *PRKAR1B* gene with highlighted CGI within promoter (green bar). **B** Data as in A for the *HEATR2* gene . **C** DNA methylation differences at *PRKAR1B*/*HEATR2* promoter assessed by pyroassay in WT (HCT116) and DKO cells (blue) and **D** SHSY5Y WT and Aza (orange) -treated cells. **E** DNA methylation levels at CG sites located within GB of *PRKAR1B* and **F** *HEATR2* covered by pyroassay in WT (HCT116) and knockout (DKO cells). Values are shown as mean +/− SD for each site: *p < 0.05; **p < 0.01; ***p < 0.001. RT-qPCR of *PRKAR1B* (**G**) and *HEATR2* (**H**) in HCT116 and SHSY5Y (**I**, **J**) cell lines. Location of pyroassays and RT-qPCR products used for this figure are indicated in Fig. 3A. Values are shown as mean +/− SD: *p < 0.05; **p < 0.01; ***p < 0.001

In addition to the significant differences in methylation at the *HEATR2*/*PRKAR1B* promoter regions noted above, we observed that the sites flanking the CGI showed high levels of methylation (Fig. 3B) and this continued further into the GB for both transcripts. Given the significant differences in promoter methylation, we considered that GB methylation here may also contribute to transcriptional activity, as seen at some other genes expressed in the brain. To investigate these possibilities mechanistically, we initially used the paired cell lines HCT116 WT (HCT116) and HCT116 DKO (DKO), as before. We first mapped the DNA methylation profiles of *PRKAR1B* and *HEATR2* in HCT116 and DKO cell lines using Illumina 450 k array and the *CandiMeth* workflow [34]. Examining a region of approximately 17 kb around *PRKAR1B* and a 13 kb region of *HEATR2* highlighted that methylation at the CGI at the promoter of both genes was even lower than in CB (max. ~ 24% in CB and ~ 4% in HCT116 WT, compare Figs. 3B and 4A). In keeping with this, no significant methylation change was observed within the promoter using pyroassay in either cell lines tested (Fig. 4C, D). However, the array data showed that there was a sharp increase in DNA methylation moving out from the CGI towards the GB in HCT116 cells, as seen in CB, indicating high levels of GB methylation across both genes. Methylation levels at array probes were substantially lower at multiple CG in the GB for both *PRKAR1B* and *HEATR2* in DKO cells (Fig. 4A, B). To confirm this, we designed new pyroassays to cover GB regions of both *PRKAR1B* and *HEATR2* as shown in Fig. 3A. These assays covered multiple CG and showed significant differences at 10/11 *PRKAR1B* CG sites and in all *HEATR2* CG sites tested (Fig. 4E, F). In agreement with these data from HCT116 cells, methylation changes at the promoter were low in SHSY5Y cells (1%) as well and not significantly affected by Aza treatment, while methylation was high in GB and decreased on Aza treatment (data not shown).

While promoter methylation did not change in either cell line, we and others have found that alterations to GB methylation can also affect transcription [8, 45]. To assess transcriptional changes at these genes in response to demethylation, we designed intron-spanning RT-qPCR assays (see Fig. 3A). While high levels of transcription of both genes could be detected in HCT116 WT cells, mRNA levels were significantly decreased (Fig. 4G, H) in DKO cell lines (*PRKAR1B p* = 0.02, *HEATR2 p* = 1.42 × 10^−08^). We also used the neuroblastoma SHSY5Y cell line to confirm our findings: both genes were significantly downregulated in the demethylated neuroblastoma cell line as well (*PRKAR1B p* = 6.33 × 10^−05^, *HEATR2* 3.37 × 10^−06^) (Fig. 4I, J). These data together showed that methylation in the GB rather than the promoter was important for facilitating expression of these genes.

### Bimodal methylation is a feature of the neurodevelopmental genes affected by FA supplementation

Given that many of the genes showing altered methylation were associated with brain (Fig. 1; Table 1) and that some of these showed a bimodal methylation pattern with low promoter and high GB methylation (above), we decided to investigate if this pattern was found more generally across the neural genes affected by folate intervention, and particularly in genes involved in higher brain function. We had already found that the 3000 top-ranking promoters and 3000 top-ranking GBs highlighted from our RnBeads analysis showed significant enrichment for loci expressed in brain (Fig. 1C). To look more closely at brain loci, we first combined promoter and GB categories and removed duplicates (Fig. 1C: (776 + 559) − 180 duplicates = 1155) to form a single list containing the names of genes whose 1) promoters or gene bodies or both showed differential methylation in our cohort and 2) which are preferentially expressed in brain. As this did not tell us what the functions of the genes were, we used the Biological Process tool in DAVID to look for particular classes which might be over-represented (Table 2). The analysis identified significant enrichment of terms related to basic brain functions such as signalling (trans-synaptic, chemical, and synaptic signalling *p* = 8.61 × 10^−17^), as could be expected given the input gene set. A number was also related to the more specific neurodevelopmental process, such as neuron development and differentiation, generation of neurons, or neurogenesis itself (*p* = 1.77 × 10^−12^–1.01 × 10^−08^). More interestingly for this cohort there were a few gene groups linked with higher-order brain function such as behaviour, learning, memory, or cognition (*p* = 3.44 × 10^−08^, 1.46 × 10^−07^, 4.09 × 10^−07^, respectively: Table 2). There were a small number of olfactory receptor (OR) genes (*n* = 8), which we and others have previously observed tend to appear as false positives in methylation screens due to being one of the largest gene families in human [47], but results were almost identical with and without these genes (shown without).

**Table 2:** Top gene ontology terms for biological process in brain-enriched group

GOTERM_BP_ALL^a^	Count	*p* value^b^	Bonferroni^c^	FDR
GO:0099536 synaptic signalling	85	2.58E−16	1.42E−12	4.04E−13
GO:0098916 anterograde trans-synaptic signalling	85	2.58E−16	1.42E−12	4.04E−13
GO:0099537 trans-synaptic signalling	85	2.58E−16	1.42E− 2	4.04E−13
GO:0007268 chemical synaptic transmission	85	2.58E−16	1.42E−12	4.04E−13
GO:0055085 transmembrane transport	131	1.63E−12	1.04E−08	2.04E−09
GO:0007399 nervous system development	187	4.01E−12	2.56E−08	4.18E−09
GO:0048666 neuron development	100	9.98E−11	6.37E−07	8.92E−08
GO:0044708 single-organism behaviour	55	1.18E−10	7.55E−07	9.24E−08
GO:0030182 neuron differentiation	118	1.62E−10	1.03E−06	1.02E−07
GO:0006811 Ion transport	134	1.64E−10	1.05E−06	1.02E−07
GO:0048699 generation of neurons	125	6.82E−10	4.36E−06	3.85E−07
GO:0022008 neurogenesis	131	7.38E−10	4.72E−06	3.85E−07
GO:0034220 Ion transmembrane transport	96	1.96E−09	1.25E−05	9.41E−07
GO:0006836 neurotransmitter transport	33	1.25E−08	7.97E−05	5.29E−06
GO:0031175 neuron projection development	83	1.27E−08	8.10E−05	5.29E−06
GO:0030001 metal ion transport	81	4.83E−08	3.09E−04	1.78E−05
***GO:0007610 Behaviour***	*62*	*4.84E−08*	*3.09E−04*	*1.78E−05*
GO:0050804 modulation of synaptic transmission	39	8.38E−08	5.35E−04	2.91E−05
GO:0007267 cell–cell signalling	127	1.43E−07	9.12E−04	4.65E−05
GO:0044707 single-multicellular organism process	387	1.50E−07	9.59E−04	4.65E−05
GO:0098660 inorganic ion transmembrane transport	73	1.56E−07	9.96E−04	4.65E−05
***GO:0007611 Learning or memory***	*33*	*1.79E*−*07*	*0.001142057*	*4.93E*−*05*
GO:0042391 regulation of membrane potential	45	1.81E−07	0.001157197	4.93E−05
GO:0030030 cell projection organization	112	2.38E−07	0.00151699	6.20E−05
GO:0007275 multicellular organism development	326	2.77E−07	0.001767572	6.93E−05
GO:0006812 cation transport	89	4.60E−07	0.002937168	1.11E−04
***GO:0050890 cognition***	*35*	*5.05E−07*	*0.003220477*	*1.17E−04*
GO:0048812 neuron projection morphogenesis	58	5.53E−07	0.003527699	1.24E−04

^a^GOTERM_BP_ALL, DAVID gene ontology database; ^b^*p* value, uncorrected *p* value; ^c^Bonferroni, corrected *p* value

**Fig. 5:**
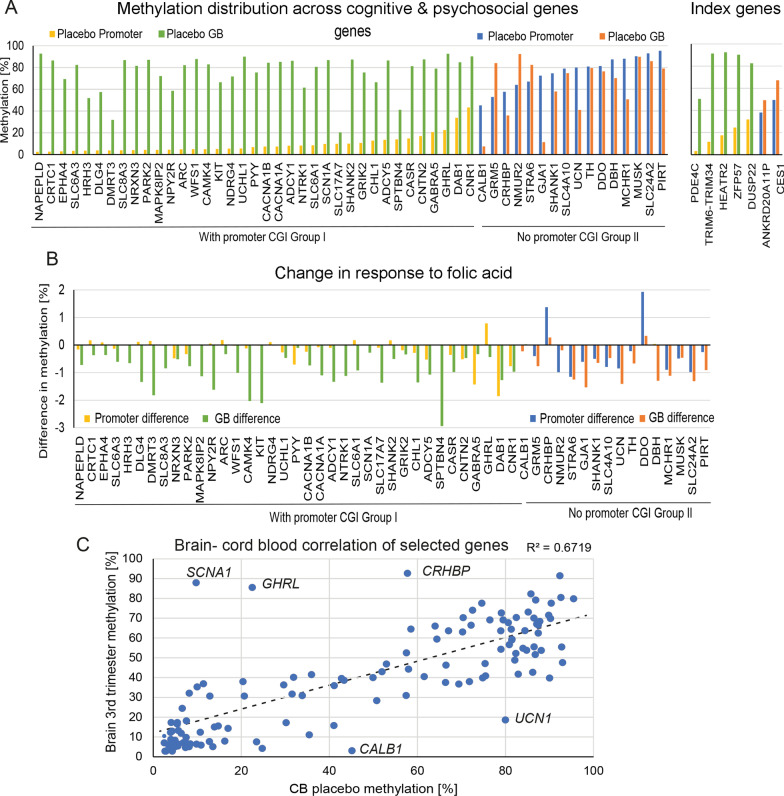
Cognitive and psychosocial genes affected by folic acid supplementation form two groups based on DNA methylation distribution. **A** Genes from the GO categories highlighted in italics in Table 2 were characterised using CandiMeth. The genes could be seen to fall into two groups based on absolute levels of DNA methylation across the promoter and GB in normal (placebo) CB. Group I (yellow/green) showed a bimodal pattern of low promoter and high GB methylation levels, often with a promoter CGI; Group II (blue/orange) were not bimodal and usually had no promoter CGI. The index genes investigated earlier are shown at right for comparison. **B** FA supplementation more often affects GB methylation among these cognitive and psychosocial genes. Mean methylation difference is calculated from the mean percentage methylation of the placebo and the FA group. **C** CB methylation levels are strongly correlated with the 3rd trimester methylation level of the gene used in this paper. DNA methylation of genes discussed in the paper compared in 2 data sets: CB (this study) and the 3rd trimester brain (GSE58885). The line of the best fit shows strong correlation (*R* = 0.746175). Note that many of the outlier genes belong to group II

Considering our previous findings indicating cognitive and psychosocial responses to FA treatment, we focused on the subgroup of 130 genes (63 genes after duplicate removal) highlighted as involved in higher-order brain function—behaviour, cognition, learning, and memory categories from Table 2 (groups highlighted in italics and bold). These genes were further analysed using our *CandiMeth* workflow on the Galaxy bioinformatics platform [34]. The workflow identified 59 genes that had probes in both promoter and GB (Fig. 5). We assessed DNA methylation at promoters vs GB as we have already observed bimodal methylation distribution in three genes marked as best ranking (Figs. 4, 5). This analysis indicated that there were two major gene groupings. The largest group (*n* = 37) showed similar DNA methylation distribution to the index genes *PRKAR1B*, *HEATR2*, and *PDE4C* with low promoter and high GB methylation (Fig. 5A, Group I yellow/green). Moreover, all genes listed in this group contain CGI within their promoter (except *DLG4*, *SCN1A*, and *CNTN2*), similar to the index genes. The second group identified did not follow this pattern, instead having more even distribution of methylation across both promoter and GB, or having high promoter and low gene body methylation (Fig. 5A Group II blue/orange), and 13/16 genes within this group do not have promoter CGI (exceptions being *GRM5*, *SHANK1*, *UCN*). Looking again at the index genes in the top 10 promoter data (Table 1), a similar split was seen, with *PDE4C*, *TRIM6-TRIM34*, *HEATR2*, *ZFP57*, and *DUSP22* having bimodal patterns (low promoter and high GB methylation), while the *ANKRD20A11P* and *CES1* genes fell into the second group (Fig. 5A, top right).

We then extended our investigation to look at the effect of FA on these genes using *CandiMeth*. For Group II genes, there was little consistency in terms of where methylation was lost or gained and subsequently the effects of supplementation are less easy to predict. Genes in Group I with low promoter–high GB methylation on the other hand lose methylation predominantly in GB following FA supplementation (Fig. 5B). This gives a clear prediction for the effects of FA on this type of gene i.e., supplementation should lead to decreases in both methylation and transcription.

One caveat of examining methylation in blood is that this may not correlate well with methylation in the brain. We therefore used publicly available data on DNA methylation in developing brain [48] and split the data into brain samples collected before 91 days (~ first trimester) and those after 91 days (~ second and third trimester—see Methods). There was a strong correlation (*R*^2^ = 0.6719) between the methylation levels of genes in CB in FASSTT and the methylation levels seen in brain during second and third trimester (Fig. 5C), with most of the genes falling along the diagonal line (the biggest exceptions being *GHRL*, *MIR648*, *SHANK*, *SLC4A10*, all from Group 2). Such a strong correlation suggests that the methylation levels obtained in the CB are comparable to those seen in brain tissue at a similar time point.

## Discussion

We have shown here that continued FA supplementation of pregnant mothers in the second and third trimester of pregnancy led to genome-wide hypomethylation in the CB of their offspring. Tissue-specific GO analysis and analysis of top-ranking regions highlighted a strong neurodevelopmental association. While there was some loss at promoters, a more marked result was the effect on GB methylation, where loss appears tied to gene repression. Here, we discuss what these overall trends may mean and the strengths and limitations of the work.

### Overall trend of hypomethylation

We confirmed here our earlier finding that supplementation lowered DNA methylation across the genome in CB for this cohort [33]. While this may seem surprising, since FA is important for production of SAM the universal methyl donor in the cell, our finding is in agreement with a number of other well-controlled studies in humans [49–52] showing mostly hypomethylation of the loci investigated. It has been suggested that a feedback inhibition loop may be triggered by increased folate concentrations in those receiving the supplement [49, 53, 54]. Increased folate levels lead to a reduction in the methylene THF, which is a substrate of the MTHFR protein and a consequential inhibition of the enzyme. Reduced MTHFR activity directly caused downregulation of the universal methyl donor SAM and alteration of the SAM/SAH ratio [54]. If such a homeostatic mechanism is occurring, it may be taking place in the foetus, where concentrations of serum folate were significantly higher than in placebo for the FASSTT cohort [32]. In contrast, supplementation served only to prevent the decrease in serum folate between GW 12 and GW 36 seen in the women receiving placebo. Preventing a drop in maternal folate levels and increasing folate concentration in foetal circulation is firmly associated with positive effects on cognitive and psychosocial outcomes both in this cohort [29–31] and in others [27, 55, 56].

### Neural gene enrichment

While modest enrichment for some neural terms, particularly those related to embryonal and neurodevelopmental forebrain pathways, has been previously seen in a FA supplementation study [49], we show here for the first time significant enrichment of genes expressed in brain, including those involved in higher brain functions such as cognition, behaviour, and learning. Additionally, we have found multiple top-ranking sites and promoters associated with brain or neurodevelopmental phenotypes. These results were particularly striking as FA supplementation in second and third trimester in this RCT has been already linked with long-lasting effects on biopsychosocial development. Children from these FA-supplemented mothers showed significantly improved cognition at 3 years of age as measured by the Bayley’s Scale of Infant and Toddler Development (BSITD-III) and better word reasoning (verbal IQ) at 7 years (*p* = 0.027) using the Wechsler Preschool and Primary Scale of Intelligence (WPPSI-III) [29]. In a separate study, they also displayed improved psychosocial development with both higher emotional intelligence, using the TEIQue-CSF, and greater resilience, using the RASP [30]. Furthermore, in our recent 11-year follow-up study, children from the FA-supplemented group performed significantly better particularly in the language domains of the Wechsler Intelligence Scale for Children [31]. In addition to the psychometric tests, we could also show that neuronal responses to language activity were significantly different using magnetoencephalographic brain imaging [31]. Thus, our current study shows concordance between improved cognitive development and performance in this cohort and altered epigenetic patterns at genes in relevant neuronal pathways.

### DNA methylation at promoter regions

The altered DNA methylation we report here affects primarily two types of genomic interval or region, namely the promoters of some genes and the GB or transcribed region of others. In addition to the zinc finger protein *ZFP57* investigated earlier [33], we showed here that the dual-specificity phosphatase *DUSP22* gene likewise gained methylation at its promoter in the FA-supplemented group, while the liver carboxylesterase *CES1* promoter lost methylation, confirmed for all three genes using pyrosequencing [33]. Our further mechanistic analysis highlighted that promoter methylation controls expression of the *ZFP57* and *CES1* promoters, though not DUSP22, highlighting the importance of functional confirmation. *CES1* is a member of the carboxylesterase family that is key in the detoxification of various xenobiotics such as cocaine and heroin [42] and may play role in the blood–brain barrier system [57, 58].

### Gene Body demethylation and repression

A second group of genes that emerged from our screen were those where high levels of GB methylation appeared to facilitate transcription. An example of this was the *PRKAR1B*/*HEATR2* locus*.* The region was initially identified as having a small (~ 3%) but significant alteration to CGI shore methylation at the promoter, verified by pyrosequencing. Further examination showed high levels of methylation throughout the GB for both genes, which is often a characteristic of highly transcribed regions. Reasoning that alterations to the shore CG may reflect changes tied to transcription, we could show in vitro that alteration of methylation at these GB resulted in decreased levels of mRNA for both genes.

*PRKAR1B* is a cAMP-dependant kinase regulatory gene highly expressed in the brain. Mutation of the 2nd exon of the gene has been found in neurodegenerative disorders such as Parkinson’s disease and novel hereditary neurodegenerative dementia involving intermediate filaments [39, 59]. Epigenetic changes occur at *PRKAR1B* during development [48], and it has been shown to be responsive to environmental changes [60, 61]. The adjacent *HEATR2* gene is essential for the function of the cilia present on some cell surfaces, as mutations are associated with primary ciliary dyskinesia [62]. The cilia are microtubule-based sensory organelles responsible for transducing environmental signals, especially during development [63, 64]. Cilia are present on some epithelia in the brain and are involved in forebrain patterning control, cerebellum development, and neural tube closure early on, but also in neuronal migration and neuronal connectivity later in gestation [65–67]. Ciliopathies commonly present with intellectual disability that can lead to hippocampal-dependent learning and memory deficits [68].

### A bimodal methylation pattern is seen on neurological targets

In addition to the enrichment for neuronal genes, a further clear pattern emerging from our analysis was that many of the genes affected had methylation levels which were low in the promoter but rose sharply and stayed high across the whole GB (*PRKAR1B*, *HEATR2*, and *PDE4C*). Following the recognition of this pattern in some of the top targets, we were able to identify two groups of genes associated with higher-order brain function that respond to FA supplementation with the aid of our *CandiMeth* workflow [34]. Group I genes were distinguished by (1) a bimodal pattern with low promoter and high GB methylation, (2) promoters overlapping CGI, and (3) alteration of methylation after FA supplementation in later pregnancy occurred in GB. Group II genes, on the other hand, (1) lacked a clear bimodal methylation distribution, (2) often had CGI within the promoter, and (3) DNA methylation alteration in response to FA occurred at the promoter region instead—examples from the top hits include *CES1*, *DUSP22*, and *ZFP57*. While methylation at the Group II genes follows the well-established paradigm of methylation-mediated promoter repression, for Group I genes methylation appears instead to facilitate transcription, since loss of methylation is accompanied by decreased mRNA levels. We have previously shown this bimodal methylation and GB methylation enhancing transcription at a group of developmentally regulated brain-specific genes in mouse [8], and indeed other mechanistic work by independent laboratories has also shown that transcribed regions of many genes are methylated at a high level and require this for normal expression [45, 69]. Recent work on DNMT3A has shown, for example, that it binds specifically to H3K36me3 [70], which is an intragenic mark of active human genes [71] and loss of DNMT3A has a repressive, not activating role, on such genes [69]. We therefore propose that such a bimodal pattern of methylation marks neuronal genes positively regulated by intragenic DNA methylation and susceptible to FA intervention.

### Methylation may mediate the relationship between folate intake during pregnancy and long-term outcomes

Given that changes in methylation in response to FA can have opposite effects on promoters and GB it is pertinent to ask what the overall outcome might be. We note that for many of the genes highlighted as affected by FA, there are reports associating detrimental outcomes with alterations of the methylation at these loci: for many of the genes for which this was the case, the changes brought about by FA were opposite in direction to those seen in the diseased state. Thus, *CES1* is hypermethylated in DS, but shows decreased methylation compared to controls in our cohort. Likewise hypomethylation of *MIR4520A*/*B* was seen after FA treatment, whereas this same region has been shown to increase methylation by a similar magnitude in young adults with depression [35]. We had previously noted that median methylation at imprinted regions also trended lower in FA-treated samples, bringing them closer to the 50% methylated status than seen in the placebo group, which were slightly elevated [33]. While these findings are only suggestive, it may be that FA supplementation helps normalise methylation levels across the genome, buffering against the decline in folate levels normally seen during pregnancy [72].

### Limitations

Here, we report a significant pattern of FA-mediated methylation changes after the second and third trimester of pregnancy, with our analysis focused mostly on neuronal and brain-related genes. The work is, however, conducted in CB samples obtained at birth, which is a peripheral tissue and may not reflect methylation levels or changes seen in the target neural tissues. As there is no similar RCT of FA supplementation analysing brain methylation that we could use to confirm our findings, we re-analysed a unique data set tracking DNA methylation changes in brain during development [48], which showed strong correlation between methylation levels of target genes in the brain and in CB, suggesting that changes observed in our CB samples could be applicable to brain as well [74]. It would be ideal to have access to tissue from brain from a RCT as well, but this is less feasible given the nature of the study: duplicating the existing trial with a larger number of participants would also be welcome. We also confirmed an important role for methylation at some of the key loci using tissue culture systems, though a limitation there is that the magnitude of the changes was greater in cultured cells. Currently, we are investigating the effects of differing FA supplementation and fortification recommendations in Spain and Canada versus the UK on methylation levels observed in CB, placenta, and saliva samples from several independent cohorts in the three countries (the EpiBrain study) which will help to increase our understanding of the correlation between folate levels in mothers and DNA methylation in offspring.

## Conclusions

We have shown clearly here that FA supplementation in later pregnancy affected the methylome of the developing child and particularly a number of brain-specific genes. Children from FA-supplemented mothers in this cohort have been shown to perform better at many cognitive tasks such as language processing, as well as having improved psychosocial development. In keeping with this, detailed analysis identified a group of genes involved in higher-order brain function such as cognition and behaviour with characteristic bimodal methylation profiles which were affected by FA treatment. Our data support and strengthen growing evidence of the benefits of continuing FA supplementation during trimesters two and three of pregnancy and its association with optimal neurodevelopment of the child.

## Methods

### Study design

The CB samples were acquired from the FASSTT study cohort, fully described in [32, 72]. Briefly, a double-blinded RCT has been conducted previously in Northern Ireland where women with singleton pregnancies who had taken FA supplements (400 μg/d) in the first trimester were recruited at 14 weeks of gestation from antenatal clinics at the Causeway Hospital, Coleraine (*n* = 226). Women were excluded if they were taking medication known to interfere with B-vitamin metabolism or if they had any vascular, renal, hepatic, or gastrointestinal disease, epilepsy, or had a previous NTD-affected pregnancy, or they withdrew from the study before randomisation (*n* = 96). Eligible participants were randomised into two groups at the end of their first trimester: one group continued to receive 400 µg/d FA (*n* = 96) and the other received placebo in pill form (*n* = 94) until the end of their pregnancy. Umbilical CB samples were collected after the expulsion of the placenta at delivery. The study was completed by 119 women, as 71 participants were excluded during the study. A total of *n* = 37 women were excluded from the FA group and *n* = 34 from the placebo group for the following reasons: participant withdrawal, pregnancy complications, prescribed FA, foetal death, non-compliance, or hospital transfer. Umbilical CB samples (45 placebos and 41 from the FA group) were collected after expulsion of the placenta at delivery, along with recording birth weight, length, head circumference, mode of delivery, and Apgar score.

### Cell culture

Human colorectal cancer cells HCT116 and DKO cells [44] were cultured in 1 g/L glucose DMEM supplemented with 10% FBS and 1 × NEAA (Thermo Scientific, Loughborough, UK). Human neuroblastoma SHSY5Y cells were cultured in DMEM/F12 medium supplemented with 10% FBS (Thermo Scientific) and treated with Aza (Sigma-Aldrich, Dorset, UK) at a final concentration of 1 μM. Treatment was renewed at 24-h intervals up to 72 h. Cells were then harvested for DNA and RNA extraction.

### DNA extraction and bisulphite conversion

DNA was extracted from CB samples or cells (HCT116 and SHSY5Y) by using the QiAMP DNA Blood Mini kit (Qiagen), according to the manufacturer’s instructions. DNA extracted from 41 FA and 45 placebo CB samples and from cells was assessed for quality control by NanoDrop 2000 spectrophotometer (Labtech International, Ringmer, UK) and by agarose gel electrophoresis. DNA from CB samples and HCT116 and DKO cells was quantified by using Quant-IT PicoGreen dsDNA Assay Kit (Invitrogen, Paisley, UK). The DNA at a concentration of 50 ng/μl was sent to Cambridge Genomic Services (Cambridge, UK), performed bisulphite conversion on the DNA in-house using the EZ DNA Methylation Kit (Zymo Research, California, USA) before hybridization to the Infinium Human Methylation EPIC BeadChip Array and scanning with the Illumina iScan according to manufacturer’s instructions (Illumina, Chesterford, UK). The genomic DNA from SHSY5Y cells was bisulphite-converted in-house using the EZ DNA Methylation Kit (Zymo Research, California, USA).

### Bisulphite pyrosequencing

In order to amplify regions of interest, primers that over span the probes with the biggest methylation change were designed using PyroMark Assay Design Software 2.0 (Table 3). Bisulphite-treated DNA was PCR-amplified using the PyroMark PCR kit; briefly, the PCR reaction was run with conditions: initial denaturation at 95 °C for 15 min; followed by 45 cycles of 95 °C for 30 s, 56 °C for 30 s, and 72 °C for 30 s; with final elongation at 72 °C for 10 min. PCR products were verified on an agarose gel before pyrosequencing which was carried out using the PyroMark Q24 (Qiagen, Crawley, UK) according to the manufacturer’s instructions.

**Table 3:** Primer sets used for pyrosequencing and transcriptional analysis used in this study, including RT-qPCR

Application	Name	Oligo sequence (5′–3′)
Pyrosequencing	pyro_MIR4520A_FW	GTTTAAATTTTTTTTTGATTTGGATAGAAA
	pyro_MIR4520A_RV	AAAACATACCCTCAATTCCAAAAAAAT C
	pyro_MIR4520A_Seq	TTTTTTTTGATTTGGATAGAAAATA
	pyro_PRKAR1b/HEATR2_Promoter_FW	TTTAGGGGTAGGTTTAGGTTTATAGT
	pyro_PRKAR1b/HEATR2_Promoter_RV	CCAACCTACCTACTAAACCTTATC
	pyro_PRKAR1b/HEATR2_Promoter_Seq	GGTAGGTTTAGGTTTATAGTT
	pyro_PRKAR1b_GB_FW	GGTTTTATAGGGATGAGGTTTTATGTATAG
	pyro_PRKAR1b_GB_RV	ACCACCCACAACATCAAACAAA
	pyro_PRKAR1b_GB_Seq	AGGTTTTATGTATAGGGTAT
	pyro_HEATR2_GB_FW	GGTAGGGAGGTATTTGGTTTAAGA
	pyro_HEATR2_GB_RV	TAAAACCCCCCCACCTACCCTTACC
	pyro_HEATR2_GB_Seq	GGGAGGTATTTGGTTTAAGATT
	pyro_DUSP22_FW	GTGAGGTTTGAGGTTAGAGATTTAG
	pyro_DUSP22_RV	ATCTCCAAATCCCCCTTTAACC
	pyro_DUSP22_Seq	TTTAGGGTAGGGAGG
	pyro_CES1_FW	GGTTGGTCGGGTTTAGTTGTTTTAAG
	pyro_CES1_RV	AAACAACACAATCCCTCTAAACTAC
	pyro_CES1_Seq	TTAAGTTTAAGTTTTAATATGGAA
RT-qPCR	qPCR_PRKAR1b_GB_FW	ACAGGGGAGTGAGGTGAGTG
	qPCR_PRKAR1b_GB_RV	TCTTTGAGGACCTGCTGGAT
	qPCR_HEATR2_FW	AGCCCTGGCGTCGTGATTAGT
	qPCR_HEATR2_RV	CCCGTTGAGCACACAGAGGCCTA
	qPCR_DUSP22_FW	CGCTGACTTGTTGACACTGC
	qPCR_DUSP22_RV	GCTCAATTGTTCCGCGTCTC
	qPCR_CES1_FW	TTGCTGCCCATGAAAACGTG
	qPCR_CES1_RV	GCATTGGAATCAACCAGCC
	HPRT_FW	AGCCCTGGCGTCGTGATTAGT
	HPRT_RV	CCCGTTGAGCACACAGAGGCCTA

Pyroassay primers are given as bisulphite-converted

### RNA extraction and transcriptional analysis

RNA was extracted from cell pellets using the RNeasy Mini Kit (Qiagen) according to the manufacturer’s instructions with an additional DNase step included degrading genomic DNA. The quantity and quality of RNA were verified by gel electrophoresis and with NanoDrop UV absorbance readings at 260/280 and 260/230 nm. cDNA was synthesized from 500 ng RNA, 250 ng random primers, 1 × U buffer RT and 200U RevertAid reverse transcriptase (all ThermoFisher Scientific). The cDNA synthesis reaction was carried out under the following conditions: 25 °C for 10 min, 42.5 °C for 50 min, and 70 °C for 10 min. RT-qPCR reactions consisted of 1 μl of 500 ng cDNA, 0.5 μM primers, and 1 × LightCycler 480 SYBR Green I Master (Roche). Reactions were carried out using the LightCycler 480 II (Roche, West Sussex, UK) using the following conditions: initial denaturation at 95 °C for 10 min followed by 50 cycles of 95 °C for 10 s, 60 °C for 10 s, and 72 °C for 10 s (elongation step). Expression was normalized to WT HCT116/SHSY5Y, *HPRT* was used as a housekeeping gene. Results were analysed using the delta-delta CT method.

### Bioinformatics analysis

Initial data processing was performed using *GenomeStudio* (Illumina v3.2). *Idat* files were analysed using the *RnBeads* package (version 1.6.1) [75] on the freely available statistical software platform *R* (version 3.1.3) and R Studio interface (Version 0.99.903). Array probes were removed during quality check, including those with a missing value (NA), probes at SNP-enriched sites, and bad-quality probes as determined by the *greedycut* algorithm, followed by the sex chromosome removal. Background correction was carried out using *methylumi.noob* [76], and the methylation values of the remaining probes were normalised using *bmiq* [77]. Also, to account for any hidden confounding variables in the data set such as maternal age, sample plate, Sentrix ID, and Sentrix Position, data were tested for association with the target variable, carried out using the *sva* package with the Buja and Eyboglu algorithm (1992), then inputting this into a *limma*-based linear model [78]. A quantile–quantile plot of observed versus expected chi-squared values was generated and showed no evidence of population substructure effects [33]. Publicly available data of foetal brain methylation analysis GSE58885 were analysed in *RnBeads* in a similar fashion. The samples were split into two groups: pre-cerebral formation (representing early pregnancy) and post-cerebral formation (representing later pregnancy) [48].

Methylation represented as *β* values were plotted against genomic loci based on Human Genome Build 19 (hg19) using GALAXY software (https://usegalaxy.org/) [79] to visualise changes in DNA methylation on the University of California at Santa Cruz genome browser (https://genome.ucsc.edu/) as described previously [80]. Differential methylation tracks and differential methylation of regions were assessed by our *CandiMeth* workflow [34]. Functional classification of enriched gene lists identified through methylation array analysis was performed using the enrichment online tool DAVID (Database for Annotation, Visualisation, and Integrated Discovery) v6.8 [37]. To assess tissue enrichment in DAVID, the UNIGENE_EST_QUARTILE database was used. A subgroup of brain-expressed genes (after olfactory genes removal) was then used for further analysis using DAVID GO TERM BP ALL database.

### Statistical analysis

All wet-lab results represent an average of two technical and a biological replicate. Differential methylation analysis was conducted onsite and region levels for controls and treated samples. On the region level (i.e. genes, promoters, CGIs), differential methylation and ranking were computed by *RnBeads* automatically based on the average difference in means across all sites in a specified region, and the quotients in mean methylation, as well as a combined *p* value, which was calculated from all site *p* values in the region using a generalisation of Fisher's method [81]. The smaller ranking number conducted for a region, the more evidence for the differentially methylated region. Results from GO analysis conducted by the DAVID enrichment tool were considered significant only when FDR < 0.05. Pyrosequencing data and RT-qPCR data were analysed using Student’s *t* test to identify statistical differences between intervention groups. A *p* value < 0.05 was considered significant. Correlation of the DNA methylation was computed by comparison of the DNA methylation levels of the mean placebo methylation value and the mean methylation of brain samples after cerebral formation (late pregnancy) from the GSE58885 data set [48]. The Pearson correlation coefficient (R^2^) was calculated by the correlation tool in Excel, where a correlation coefficient of + 1 indicates a perfect positive correlation and a correlation coefficient near 0 indicates no correlation.

## Supplementary Information

**Additional file 1. Fig. S1**: FA increases promoter methylation of *DUSP22* and decreases methylation of micro-RNA *MIR4520A/B.* a Genome browser UCSC schematic of DNA methylation difference within the promoter of *DUSP22* gene obtained by EPIC array (top track presenting blue bars increase, red bars—decrease in methylation). UCSC RefSeq gene construction is shown in blue. The position of pyroassay product for methylation and RT-qPCR product for accessing expression of the gene is shown in black. Δβ, mean difference in β value between placebo and FA-treated groups; maximum gain and loss also shown (+ 0.11β = 11%, - 0.13β = 13% methylation). b Methylation increase in *DUSP22* promoter as a response to FA treatment in CB samples analysed by EPIC array showing the specific CG sites corresponding to the pyroassay product shown in a. c Comparison of two methylation analyses EPIC array and pyroassay showing a difference in methylation at the *DUSP22* promoter between placebo and FA-treated CB samples. Components of the table same as Fig. 2D. d Methylation levels at individual CG sites covered by the pyroassay in WT (HCT116, blue) and knockout (DKO, orange) cells. Values are shown as mean +/− SD for each site: *p < 0.05; **p < 0.01; ***p < 0.001. e RT-qPCR showing no change in transcription of the gene in HCT116/ DKO cells using the primers indicated in a, values normalized to *HPRT*. f Genome browser UCSC schematic of DNA methylation difference within the *MIR4520A/B* obtained by EPIC array (top track presenting red bars- decrease in methylation). UCSC RefSeq gene construction is shown in blue. The position of the pyroassay product for methylation of the gene is shown in black. Δβ, mean difference in β value between placebo and FA-treated groups; maximum loss also shown (- 0.11β = 11% methylation loss). g Significant methylation decrease in *MIR4520A/B* as a response to FA treatment in CB samples analysed by EPIC array and confirmed by pyrosequencing within the *MIR4520A/B* region covering the CG site corresponding pyroassay product shown in f. h Comparison of two methylation analyses EPIC array and pyroassay showing loss in methylation of the *MIR4520A/B* (location is shown in Fig. S1f) between placebo and FA-treated CB samples.

**Additional file 2. Table S1**: Top 10 sites with differential methylation in response to folic acid also show neurodevelopmental associations. **Table S2**: Top 10 ranking gene body regions showing differential methylation.

## Data Availability

The data sets used and analysed during the current study are available under GSE176325.

## Abbreviations

Aza: 5-Aza 2-deoxyazacytidine
BSITD-III: Bayley’s Scale of Infant and Toddler Development
CB: Cord blood
R: Correlation coefficient
CGI: CG island
CG: Cytosine–Guanine site
DAVID: Database for Annotation, Visualisation, and Integrated Discovery
DNMTs: DNA methyltransferases
DS: Down’s syndrome
FDR: False discovery rate
FASSTT: Folic acid supplementation in second and third trimester
FA: Folic acid
GB: Gene bodies
GO: Gene ontology
GW: Gestational week
DKO: Double knockout
NTDs: Neural tube defects
OR: Olfactory receptor
pyroassay: Pyrosequencing assay
RCT: Randomised controlled trial
RASP: Resiliency Attitudes and Skills Profile
SAM: S-adenosylmethionine
SNP: Single-nucleotide polymorphism
TEIQue-CSF: Trait Emotional Intelligence Questionnaire Child Short Form
WPPSI-III: Wechsler Preschool and Primary Scale of Intelligence
WT: Wild type
ZFP57: Zinc finger protein 57
