# Supplementary figures and images for: Folic acid intervention during pregnancy alters DNA methylation, affecting neural target genes through two distinct mechanisms
Source: Clin Epigenetics. 2022 May 16;14:63. doi: 10.1186/s13148-022-01282-y (PMC9112484; doi:10.1186/s13148-022-01282-y)

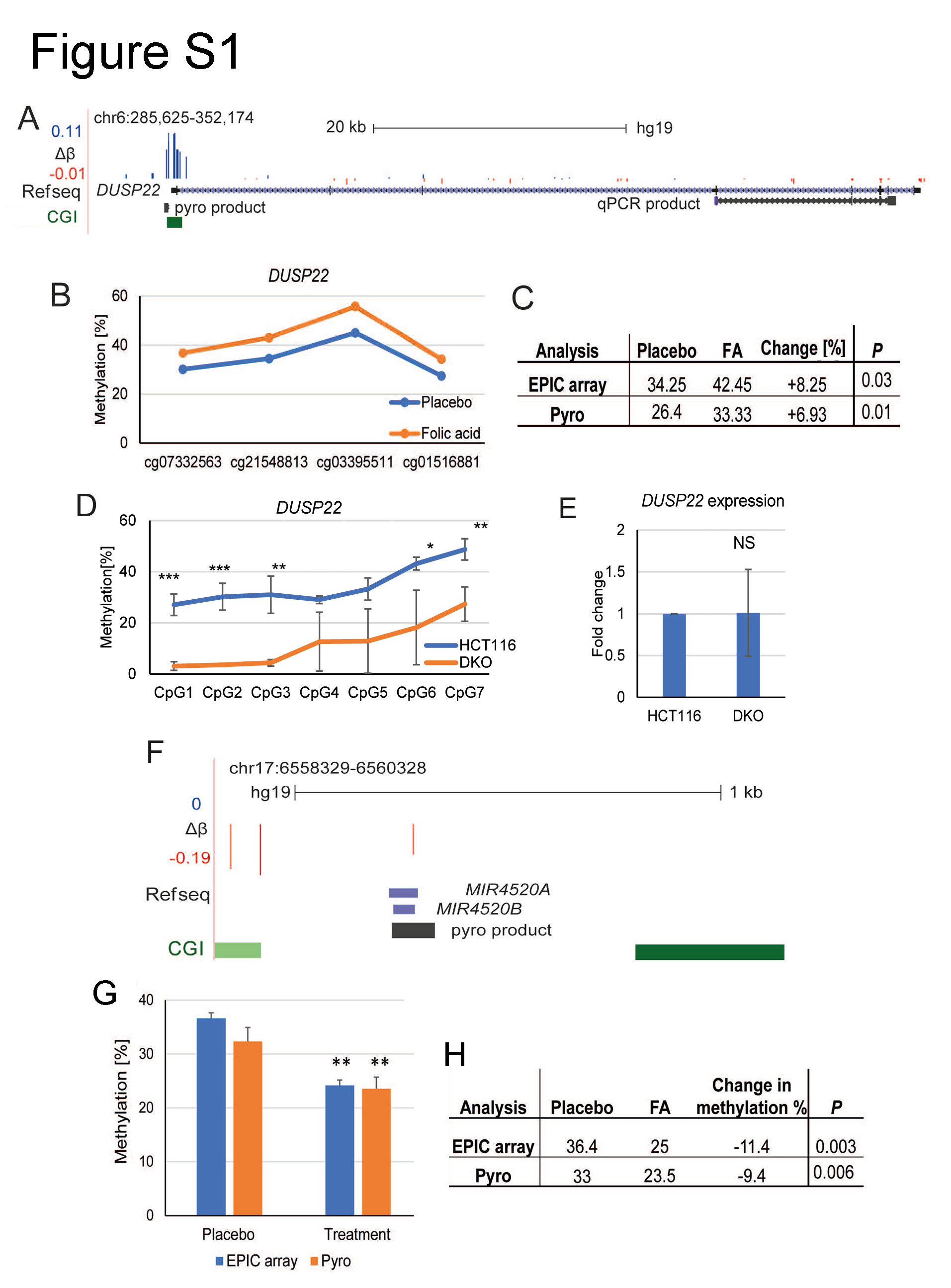

Supplement: Supplementary file 1 — Additional file 1. Fig. S1: FA increases promoter methylation of DUSP22 and decreases methylation of micro-RNA MIR4520A/B. a Genome browser UCSC schematic of DNA methylation difference within the promoter of DUSP22 gene obtained by EPIC array (top track presenting blue bars increase, red bars—decrease in methylation). UCSC RefSeq gene construction is shown in blue. The position of pyroassay product for methylation and RT-qPCR product for accessing expression of the gene is shown in black. Δβ, mean difference in β value between placebo and FA-treated groups; maximum gain and loss also shown (+ 0.11β = 11%, - 0.13β = 13% methylation). b Methylation increase in DUSP22 promoter as a response to FA treatment in CB samples analysed by EPIC array showing the specific CG sites corresponding to the pyroassay product shown in a. c Comparison of two methylation analyses EPIC array and pyroassay showing a difference in methylation at the DUSP22 promoter between placebo and FA-treated CB samples. Components of the table same as Fig. 2D. d Methylation levels at individual CG sites covered by the pyroassay in WT (HCT116, blue) and knockout (DKO, orange) cells. Values are shown as mean +/− SD for each site: *p < 0.05; **p < 0.01; ***p < 0.001. e RT-qPCR showing no change in transcription of the gene in HCT116/ DKO cells using the primers indicated in a, values normalized to HPRT. f Genome browser UCSC schematic of DNA methylation difference within the MIR4520A/B obtained by EPIC array (top track presenting red bars- decrease in methylation). UCSC RefSeq gene construction is shown in blue. The position of the pyroassay product for methylation of the gene is shown in black. Δβ, mean difference in β value between placebo and FA-treated groups; maximum loss also shown (- 0.11β = 11% methylation loss). g Significant methylation decrease in MIR4520A/B as a response to FA treatment in CB samples analysed by EPIC array and confirmed by pyrosequencing within the MIR4520A/B region covering t [file 13148_2022_1282_MOESM1_ESM.tif]
