# Supplementary material for: Folic acid intervention during pregnancy alters DNA methylation, affecting neural target genes through two distinct mechanisms
Source: Clin Epigenetics. 2022 May 16;14:63. doi: 10.1186/s13148-022-01282-y (PMC9112484; doi:10.1186/s13148-022-01282-y)
Supplement: Supplementary file 2 — Additional file 2. Table S1: Top 10 sites with differential methylation in response to folic acid also show neurodevelopmental associations. Table S2: Top 10 ranking gene body regions showing differential methylation. [file 13148_2022_1282_MOESM2_ESM.docx]

## Table S1 Top 10 sites with differential methylation in response to folic acid also show neurodevelopmental associations

| **Sites/ gene^1^** | **CG id^2^** | **Chr. ^3^** | **mean diff [%]^4^** | **Rank^5^** | **diffmeth. p.val^6^** | **Function** | **Reference** |
| --- | --- | --- | --- | --- | --- | --- | --- |
| [ATP11A] | cg21463262 | chr13 | -23.12 | 150 | 0.00021 | SNP missed by initial quality control | - |
| **PRKAR1B** | cg06242242 | chr7 | -8.97 | 275 | 0.00027 | Expressed in brain, mutation causes neurodegenerative disorders | ENSG0000188191.10 expression from GTEx [39] |
| NXN | cg08104960 | chr17 | -8.94 | 280 | 0.000381 | Cell growth and differentiation, Robinow Syndrome | RefSeq, Sep 2015, [89] |
| **PRKAR1B** | cg05729249 | chr7 | -7.51 | 496 | 0.000173 | [see above] | - |
| [MAGI2] | cg00401665 | chr7 | -9.85 | 499 | 0.000647 | SNP missed by initial quality control | - |
| NXN | cg17030231 | chr17 | -8.96 | 600 | 0.000675 | [see above] | - |
| RAD51B | cg21123519 | chr14 | 7.04 | 607 | 0.000695 | Involved in homologous DNA pairing, immune response | [41,90] |
| AK125858 | cg15695738 | chr19 | -7.04 | 877 | 0.001144 | - | - |
| CYP4V2 | cg24794857 | chr4 | -7.42 | 941 | 0.001234 | Metabolism of fatty acid, Bietti crystalline corneoretinal dystrophy | [91,92] |
| **SEMA4F** | cg25608392 | chr2 | -9.13 | 997 | 0.001316 | Neural development, expressed in nervous system, glial cells | [40,93,94] |
| *^1^genes with neural associations in bold, single nucleotide polymorphism (SNP) missed by RnBeads in square brackets; ^2^CG id, identity number of the CG probe on the EPIC array; ^3^Chr, chromosome; ^4^mean.diff [%], difference in mean β value expressed as %, ^5^Rank, RnBeads computed ranking value (lowest being best); ^6^diffmeth.p.val, differential p value.* | | | | | | | |

## Table S2 Top 10 ranking gene body regions showing differential methylation

| **Gene^1^** | **Chr. ^2^** | **mean. diff [%]^3^** | **Rank^4^** | **comb.p.val^5^** | **Function** | **Reference** |
| --- | --- | --- | --- | --- | --- | --- |
| **MIR4520A; MIR4520B** | chr17 | -12.25 | 36 | 0.007579704 | Hypermethylated in young adults with depression | [35] |
| MIR648 | chr22 | -3.99 | 55 | 0.009688025 | - | - |
| [PTCHD3P2] | chr2 | -3.73 | 78 | 0.011778394 | Pseudogene | - |
| FOXD4L5 | chr9 | -2.81 | 117 | 0.016379896 | DNA binding | (GeneCard) |
| [USP32P2] | chr17 | -5.59 | 172 | 0.028172129 | Pseudogene | - |
| MIR4740 | chr17 | -3.45 | 189 | 0.029751513 | - | - |
| **ZFP57** | chr6 | 3.78 | 196 | 0.030266206 | Imprinted gene regulator, imprints expressed in brain | [82,83] |
| [ALOX15P2] | chr9 | -2.58 | 233 | 0.018118707 | Pseudogene | - |
| [ZNF726] | chr19 | -1.79 | 320 | 0.043659484 | Pseudogene nucleic acid binding and DNA-binding transcription factor activity | (GeneCard) |
| [IMMTP1] | chr21 | -3.97 | 384 | 0.053429621 | Pseudogene | - |
| *^1^genes previously investigated are in bold, pseudogenes in square brackets; ^2^Chr, chromosome; ^3^mean.diff [%], difference in mean β value expressed as %, ^4^Rank, RnBeads computed ranking value (lowest being best); ^5^comb. p val, combined p value.* | | | | | | |

# 
